# Hospital and Institutional News

**Published:** 1912-05-11

**Authors:** 


### HOSPITAL AND INSTITUTIONAL NEWS.

### HOSPITALS AND HOSPITAL FUNDS.

The action of King Edward's Hospital Fund and of the Metropolitan Hospital Sunday Fund in regard to St. George's Hospital formed the subject of a leading article in the *Lancet* of April 20 and in *The Times* of May 2. We dealt fully with the questions at issue in The Hospital of March 16, pages 596 and 613-14. The *Lancet* article is a well-reasoned, moderate and interesting statement, which has been written by someone who is not wholly familiar with the points at issue. The article in *The Times* presents a striking contrast to the *Lancet's* contribution to the subject from the fact that it contains remarkable inaccuracies, and is a departure from the well-established practice of *The Times* by failing to be judicial and impartial throughout. *Ex parte* statements in a leading article in *The Times* constitute a new departure which we hope may not be regarded as a precedent. In our article on March 16 we demonstrated clearly that the points of difference between the King's Hospital Fund and St. George's Hospital can be happily determined by St. George's Hospital repaying to the general fund the sums wrongly paid in 1909 and 1910 out of the general funds to its medical school. In this way—and it is the only right way—St. George's Hospital at once can place itself in the position to apply for a grant from the King's Hospital Fund, and to base its claim for it upon the circumstances that this step has been taken, that in future the authorities will enter in the uniform system of accounts their discretionary fund, as they did formerly, and that they will henceforth follow the course adopted by all other hospitals with medical schools which receive a grant from the King's Fund. It follows that only so far as the questions directly affect St. George's Hospital can they have any real interest for the voluntary hospitals of London and for the public who support them.

### THE FLORENCE NIGHTINGALE MEMORIAL.

We have great pleasure in reporting that since The Hospital actively interested itself in this memorial, with Lord Pembroke's zealous co-operation and help, the subscriptions have increased from some £2,000 to over £8,000. Of this sum £4,000 has been assigned to the Nightingale Annuities Fund, of which Lord Pembroke and Messrs. Coutts & Co., the bankers, will be the trustees. Lord Pembroke's trusteeship will extend to his successors and so become a sacred trust in his family, who can thus take an active interest in the granting of the annuities and in the annuitants as well as in the safe custody of the capital funds. The Trained Nurses' Annuity Fund have been invited to undertake the executive work connected with the allotment of annuities and the incidental payments in connection therewith. A number of regulations as to grants have been adopted by the Executive Committee, and to be eligible every candidate must hold a certificate of full training in some recognised hospital with a training school which will satisfy the Council of the Trained Nurses' Annuity Fund. Mr. Arthur G. Walker, of Cedar Studios, Chelsea, who has done some very beautiful work, has been unanimously entrusted with the commission as sculptor to execute the bronze statue of Miss Florence Nightingale. We hope that as the funds are sufficient arrangements will be made to place a replica of the statue of Florence Nightingale with a suitable inscription in Westminster Abbey, and we should hope also in St. Paul's Cathedral. This would ensure to the fullest possible extent the perpetuation of the spirit of Florence Nightingale in the work of nursing throughout the Empire. The details of the inscription and accompanying groups to be placed on the base of the statue have, we believe, yet to be approved. A strong desire has been expressed that the names of the first group of nurses who accompanied Florence Nightingale to the Crimea should be inscribed on the pedestal.

### A SANATORIUM FOR BANKERS.

The Council of the Institute of Bankers has accepted an offer from Wernher, Beit and Co. of the London Open-air Sanatorium, Pinewood, Wokingham, Berkshire. The sanatorium is offered complete and fully equipped for the benefit of officers and clerks of the banking and financial businesses, not merely of London but of the provinces. Eleven years ago Wernher, Beit and Co. started to maintain this institution, which the firm had built, and which was designed for "the treatment of cases of consumption among the educated middle class" at three guineas a week. The Council of the Institute of Bankers has received such satisfactory guarantees against loss in maintaining the sanatorium that what was an institution for the middle classes maintained by a firm of philanthropic bankers has become an institution for bankers maintained by that business itself. It may be interesting to recall that the sanatorium has accommodation for 64 patients, grounds of 82 acres on the Bagshot sands, and that the medical staff consists of two resident officers. What may be called the sanatorium movement has produced many forms of co-operation. One of the most valuable is that by which individual businesses have co-operated to provide open-air treatment for their phthisical employees.

### SHORTER HOURS FOR A MENTAL HOSPITAL STAFF.

The attendants at Cardiff Mental Hospital have won the greater part of their petition, which we noted last week, for improved working conditions. The special committee which was appointed to consider the matter has since met and decided, subject to the sanction of the City Council, to grant one day's rest in seven for the day staff, one night's rest in seven for the night staff, and money in lieu of rations at the rate of 1s. 5d. per diem for the married staff on each weekly day's leave. The request for money in lieu of rations during the annual leave was not granted. The effect of the concessions will be to increase the staff by eleven, and their cost, including the money grant for the weekly day off, has been estimated by Alderman Morgan Thomas at £680 a year. Hitherto the staff has worked on an average 77¾ hours a week in summer and 80 in winter. The agitation for a reduction of hours in mental hospitals is, of course, an old one, and the one day's or one night's rest in seven now granted will mean a 72-hour week. Dr. Goodall, the medical superintendent, has inquired of twenty-one mental hospitals, and thirteen of these have granted this concession. He reminded the meeting that a Bill had been promoted in Parliament to limit by statute the hours of mental hospital staffs to 60 a week for day and night attendants, but a Select Committee has recommended that this number should apply to the night staff only, and that the week should be 70 hours for the day staff. There is no doubt that the strain of mental work is very great, and that a day or night's rest in seven should be the practice in every, as it now is in the majority, of mental hospitals.

### MENTAL HOSPITAL STAFF APPOINTMENTS.

A number of staff changes at the various London mental hospitals are announced. At Banstead Mental Hospital Mr. M. W. Ruthven has resigned the post of sixth assistant medical officer, and Mr. M. J. McGrath has been appointed to succeed him. Mr. E. S. Litteljohn, third assistant medical officer at Hanwell Mental Hospital, has been transferred to be second assistant medical officer at Cane Hill Mental Hospital. He succeeds Dr. Norcliffe Roberts, who has been transferred to Horton Mental Hospital. Mr. I. J. Roche, third assistant at Cane Hill, has resigned his appointment. Mr. J. C. Wootton and Mr. F. Morres, the fourth and fifth assistants, have been promoted each a grade, and Mr. Dominick O'Flynn has been appointed fifth assistant. Consequent on Mr. Litteljohn's transfer to Cane Hill, the fourth, fifth, and sixth assistant medical officers at Hanwell Mental Hospital have been promoted each one grade, and Mr. G. W. B. James has been appointed to be sixth assistant. At the Horton Mental Hospital Miss P. E. Smith has resigned her appointment as matron. The salary of Dr. M. A. Collins, the medical superintendent at the Epileptic Colony, has been increased from £615 to £650 a year, and the salary of Miss Hoffman, the matron, has been increased from £93 to £95 a year, rising by two annual increments to a maximum of £100 a year.

### A MAYOR AND THE MENTAL HOSPITALS COMMITTEE.

The Mental Hospitals Committee of the London County Council has recently elected Mr. W. Whitaker Thompson to be its vice-chairman during the ensuing year. Mr. Thompson, who takes a deep interest in the committee's work, is an indefatigable member of the London County Council. At the present time he is Mayor of Kensington, and he is a member of several committees of the County Council.

### FIRST MEETING OF THE SECRET REMEDIES COMMITTEE.

The first meeting of the Select Committee on Proprietary Medicines was held at the House of Commons on Thursday last week, and its main object was to decide upon the method of procedure. Consideration will first be given to the existing law applicable to the sale of secret medicines in this country. Following on this, evidence will be adduced with reference to the law in foreign countries and the British Dominions. The inquiry is likely to be thorough, and to last for some considerable time. Most of the interests concerned have appointed "watching" committees.

### THE HOUSING OF THE ROYAL SOCIETY OF MEDICINE.

The presence of the King and Queen at the coming opening ceremony of the new house of the Royal Society of Medicine is an eloquent sign—if sign were needed—of the appreciative interest which their Majesties take in the problems of the day. The Royal Society of Medicine represents the head-quarters of the army that is fighting the battle against disease. It does not, it is true, dictate to the various arms and branches of the service exactly what they shall do, as the commander-in-chief of an army does; but it at least receives the reports of their progress from the many corps of specialists, and it often co-ordinates the work of two or more of such separate departments. When the ambitious scheme for moving from Hanover Square was first launched there were many criticisms offered. Some of these were directed against the whole principle of moving and rebuilding; others against the site selected and the building scheme adopted. Nor can it be said that the criticisms have been entirely silenced as yet. It remains for those who advocated and carried through the proposal to show by the future work and efficiency of the Royal Society of Medicine in its new house that the change and expense were justified. The recent history of the Society is one of remarkable activity, and the prospects for the future are equally bright; its dignity cannot fail to be enhanced by the splendid new premises now to be inaugurated.

### A SUPPOSED DANGER IN THE LACE TRADE.

The recent official announcement that the Home Secretary has appointed a Committee to report whether certain diseases, one of which is Dupuytren's contraction, should be added to the list of industrial diseases for which compensation is payable by law, must have surprised most people who read it. Dupuytren's contraction is a peculiar condition which affects especially the ring and little fingers of each hand, drawing them down into the palm, so that in the end they become useless. It rarely begins in patients under forty, and usually progresses so slowly that it may be years before any great inconvenience is caused. At one time it was thought that gout had something to do with its origin. It is more common in men than women, and is found in all classes of society. It appears that this complaint is common among the Nottingham lace hands; according to one assertion 4 per cent. of the twisthands suffer from it. It is this which has prompted the reference to the Committee, and it will be of interest to note what conclusions they arrive at concerning the matter. It is supposed that the pulling of the heavy levers of the lace machines may be the exciting cause of the contraction. If this be the case one might expect signalmen to suffer from it, and in any case it will hardly explain its occurrence in brain workers, for at the present time a fairly well-known London consultant suffers from it, and he has never been a lacemaker.

### THE AVERAGE YIELD OF A LINEN LEAGUE.

As it is sometimes asked by those who intend to help newly-built hospitals by founding a linen league what is a fair year's work, we take the year's record of the linen league attached to the Royal Bucks Hospital, Aylesbury, as an example. Within the last twelve months the league has supplied 345 articles, besides which it has undertaken to provide twelve pairs of blankets, twelve sheets for private patients, and twelve pillow-slips. Lady Louisa Smith, who has been re-elected President, said that this was a fair year's work; and read in the light of the size of the hospital and the length of time which the league has been established, the figures have a significance which other organisations should not miss.

### PUBLIC VACCINATORS AND SUPERANNUATION ALLOWANCES.

A medical practitioner, who until lately has been medical officer and public vaccinator to a Wiltshire Board of Guardians, after having been compelled through ill-health to resign, has brought an action against them, claiming to be entitled to a superannuation allowance on the ground that he was an officer in the employ of the Guardians within the meaning of the Act of 1896. The Guardians admitted that he was entitled to a superannuation allowance as medical officer of health, but denied his claim to such an allowance as public vaccinator. Their point was that the two posts were quite distinct, and that vaccination fees were not "emoluments" within the meaning of the Act of 1896. The Guardians' view was upheld, and the superannuation allowance of the plaintiff, as medical officer of health, was agreed to. The decision, no doubt, will be a disappointment to the many medical officers of health throughout the country who supplement their incomes by the fees they obtain in their additional capacity as public vaccinators.

### THE NEW SECRETARY OF THE BRITISH MEDICAL ASSOCIATION.

The Council of the British Medical Association has appointed Dr. Alfred Cox, M.B., to be medical secretary. Dr. Cox, who has been deputy medical secretary since 1908, has been acting medical secretary for the last five months. He represented the North of England branch on the Parliamentary Bills Committee from 1899-1901, and on the Central Council, 1902-03, and was its President in 1907-08. He was also on the committee which drafted the scheme of reorganisation ten years ago. Dr. Cox, who is a graduate of Durham University, has published in the medical Press various papers, including the "Financial Aspect of Contract Practice."

### CHARGES AGAINST AN ISOLATION HOSPITAL.

Soon after this issue is in the hands of our readers a special meeting of the Hinckley Isolation Hospital Committee will be held to investigate certain serious charges which have been preferred against the staff. Briefly, it is alleged by the mother of three girls who were recently admitted to the Isolation Hospital with scarlet fever, that the eldest was given bandages to wash which were infected with eczema, and from which she caught it herself; that the same daughter had to sit up with a dying girl till 11 P.M.; that her two sisters were sent home with unclean heads; and that the youngest arrived with sores on her feet, alleged to have been caused by filing. To these charges Miss Myra Forsyth, the matron, has already given a detailed reply; its broad basis being that there was very little foundation for any of the allegations. Incidentally, however, the matron's report stated that the wards were much overcrowded, and that "fully 90 per cent. of all cases admitted" are as regards the heads in a verminous condition. It appears also from the statement of Dr. J. A. T. Hall, the medical officer, that an abnormal number of cases has been admitted during the past twelve months. Pending the result of the inquiry we, of course, refrain from comment.

### A POOR-LAW INFIRMARY LEFT IN THE LURCH.

The Sheffield Board of Guardians is experiencing considerable difficulty in obtaining a hospital medical staff. Dr. J. Hewat, of Lodge Moor Hospital, who was recently appointed assistant medical officer, refused to take up the appointment on the ground that its conditions had been changed. Almost simultaneously Dr. E. R. Flint, of Leeds, who was appointed senior medical officer, cancelled his appointment, apparently to apply for another post. The clerk has been asked to express the dissatisfaction of the hospital committee at this action. These events have lead to the promotion of Dr. Balfour M'Kean, who was appointed junior assistant at the time of Dr. Flint's selection, to the senior assistant post, while Dr. Branford Morgan, house surgeon at the Sheffield Royal Hospital, has been appointed junior resident assistant medical officer.

### A PARISIAN CURE FOR HOSPITAL ABUSE.

Paris apparently has taken very drastic measures to prevent the abuse of hospitals by patients who can afford to pay. According to an American contemporary, proceedings have been initiated by the director of a Paris Eye hospital against a well-to-do patient who obtained free treatment for cataract. The Oculists' trade union discovered his means and protested to the hospital, with the result that the patient, to avoid prosecution, paid the £40 damages which the Minister of the Interior had authorised the institution to demand. Half of this sum has been handed to the Oculists "in recognition of the injury done to the medical profession." An important precedent has now been created, which, if followed, will, it is hoped, be sufficiently severe to prevent well-to-do patients from seeking free hospital treatment.

### A HOSPITAL PRESIDENT DIES IN HARNESS.

Mr. John Dodd, J.P., the president of the Oldham Infirmary, who died recently, had held the post for seven years. Other claims upon his time—he was the head of a well-known industrial firm—and failing health sometimes prevented his attendance at meetings, but an example of the pains which he took on behalf of the hospital is worth recording. The last day of his life he spent in going from place to place examining different types of machinery, so as to advise the committee which would be the best to place in the new laundry.

### £135,000 FOR CHARITY.

Mrs. Elizabeth Rebecca Johnson, of Bournemouth, whose charitable bequests amount to the above sum, which represents approximately half her net estate, has left the greater part of it to hospitals. We note particularly £5,000 each to the Radcliffe Infirmary, Oxford, and to the Royal Berkshire Hospital, Reading; £4,000 each to the Victoria Hospital, Bournemouth, the Gore Farm Convalescent Hospital, Dartford, Kent, and the National Anti-Vivisection Hospital, Battersea Park, S.W.; £3,000 to the Royal Hospital for Incurables, West Hill, Putney; £2,000 each to the Bournemouth Sanatorium for Diseases of the Chest, the Boscombe Hospital, the Royal National Hospital for Consumption, the Waterloo Hospital for Children and Women, the National Hospital for Paralysis, Soho Square, W., the British Home and Hospital for Incurables, the Northern Convalescent Hospital, Winchmore Hill, N., the Infirmary for Consumption, Margaret Street, W., the Free Home for the Dying, Clapham, the Wimbledon Hospital for Infectious Diseases, the Royal Free Hospital, the Central London Ophthalmic Hospital, and the National Anti-Vivisection Society; £1,000 each to the North-Eastern Fever Hospital, South Tottenham, the Royal Hospital, Chelsea, the Nottingham General Hospital, the Dorset County Hospital, the West End Hospital, and the Invalid Children's Home, Church Fields, Margate; £500 each to the Chelsea Hospital for Women, the London Temperance Hospital, Hampstead Road, N.W., and the Weymouth Eye Infirmary. Mrs. Johnson stated in her will that she desired to be buried in her husband's tomb at Bournemouth, and "I wish my funeral to be conducted liberally and regardless of expense," a provision which is not usually found in a will which gives so much evidence of public spirit.

### PERTH'S NEW INFIRMARY.

Almost exactly a year ago the first sod of Perth's new infirmary was cut, and last August took place the laying of the memorial stone by Mrs. Charles A. Murray, of Taymount. Since then the work has gone on steadily, and already the out-patient and administrative block, operating theatre, and the two lodges are roofed. The concrete floors are laid in the out-patient admission block and theatre and in a large section of the main corridor. The main surgical block is also roofed and most of the concrete floors in this block are laid. The walls of the administrative block are built to main-cornice level and ready for roofing, as is also the kitchen block. The main medical block has the walls built to the first-floor level, and this, along with the other portions of the building, is being rapidly pushed forward, and, should the weather prove favourable, it is expected that the entire buildings will be roofed in and a large proportion of the internal finishings in position by the end of this month. The hospital is to accommodate 136 patients.

### DIET IN SCHOOLS.

On Monday next a Conference on Diet in Schools will be held at the Guildhall under the presidency of the Lord Mayor, who is demonstrating almost daily the advantages which a medical chief magistrate can confer upon the City and the Country. This conference is organised by the National Food Reform Association, and it comes so pat upon last week's scholastic libel case that it may gain thereby a measure of public attention larger than it otherwise would, though not larger than it deserves. There is no doubt at all in the minds of those best qualified to judge that large numbers of children at boarding schools are not fed so liberally as they ought to be. Curiously enough, these shortcomings are especially reported from the most expensive schools, where there is least excuse for them. Individual medical officers have here and there protested, but too many of them are muzzled by the necessity of retaining the goodwill of the house masters. Nevertheless, public opinion is beginning to take cognisance of and to condemn this state of things, and, indeed, when the children in the elementary schools are fed at the expense of the ratepayers, it does seem too absurd that boys at first-rate public schools should not get enough. The evil only requires ventilation and discussion to be remedied; both these it will obtain at the Guildhall, and the proceedings will be followed with close attention both by parents and by the medical profession.

### NEW WARD AT THE NEWARK HOSPITAL.

The Duke and Duchess of Portland visited Newark Hospital last Saturday. The ceremony of opening the new children's ward was performed by the Duchess, who received purses from the children. It is chiefly owing to further contributions from the workmen and children of Newark and the district that it has been found possible to open the ward. Refreshments were served in a large tent to 100 workmen representatives and 100 children with their teachers. Mr. Davy, the chairman, afterwards conducted the Duke and Duchess round the hospital, accompanied by Dr. Heard, house surgeon, and Dr. Hallowes. The Duchess spoke to each patient, sister, and nurse. Every effort was made to consolidate local support, and it is hoped that the new children's ward thus inaugurated will be well maintained by the working-class families, who already have worked so well to secure its opening.

### IMPROVEMENTS DECIDED ON AT BEDFORD.

The medical staff of the Bedford County Hospital is to be congratulated upon the £1,000 which the board of management has agreed to expend in providing a new *x*-ray room and electrical apparatus. As we have already pointed out, the medical staff was very anxious for this improvement, though there was some thought of devoting the money to laundry alterations. Mr. G. C. Walker hopes that these also may be carried out before very long. By the way, an important change in the staff has occurred by the retirement of the matron, Miss Munro, who for fourteen years, in the words of the report, has presided over the destinies of the institution. The election of Mr. Beauchamp Wadmore, the recently appointed secretary, has been confirmed.

### THE FIRST CHINESE DOCTOR TO BE KNIGHTED BY THE KING.

The honour of knighthood bestowed by the King on Dr. Ho Kai, C.M.G., at the recent opening of the Hongkong University is a somewhat notable event. Not only is Dr. Ho Kai the first Chinese British subject to receive the honour of knighthood, but he is the first Chinese graduate in medicine to be thus distinguished. Those who have the privilege of the new knight's acquaintance can gladly testify to the highly meritorious career of Sir Kai Ho Kai. He was educated at Palmer House School, Margate, and Aberdeen University, where he graduated M.B., and subsequently studied for the Bar, becoming Senior Equity Scholar in 1881, and has practised in Hongkong since 1882, after admittance to the Bar in that Colony. He was also married in England. It was in memory of Mrs. Ho Kai, who died in Hongkong in 1884, that he subsequently founded the Alice Memorial Hospital in Hollywood Road, Hongkong, where Chinese can receive treatment on Western methods. When the Hongkong College of Medicine for the Chinese was founded a few years later it found a home in the Alice Memorial Hospital, and Dr. Ho Kai joined the governing body; he has ever since taken the warmest interest in its progress. For years he has acted as Lecturer on Medical Jurisprudence and Rector's Assessor. He was appointed member of the Legislative Council in 1885, and has now for several years been senior unofficial member of that body. He was for ten years a member of the Sanitary Board, and has served on a great number of Government committees, and been identified with every movement for the benefit of the Chinese community and the good of the Colony almost continuously for more than a quarter of a century. There was one little break in this connection during the nineties, when Dr. Ho Kai was induced to take service under the Chinese Government. He thought he might be useful to his fellow-countrymen by reason of his foreign training and his long experience of Western ways and methods, as well as his knowledge of Western law. But he soon found that the Chinese officials only desired to utilise his influence with the Cantonese in a direction not calculated to promote their interests. In 1902 he was made a Companion of the Order of St. Michael and St. George.

### AN OLD CITY CHARITY.

Under this heading in our issue of April 27, as Mr. R. A. Owthwaite has properly pointed out, less than justice was done to the City of London Lying-in Hospital, which contains 60 beds and so is one of the largest of lying-in hospitals. We regret the inadvertent error on this latter point which was made in our issue of 27th ultimo. It is a pleasure to see Mr. Owthwaite's handwriting once again, for he is in fact one of the oldest of London hospital secretaries on the active list, and has done yeoman service in the cause of all the hospitals and hospital management, generally, by having acted for so many years as statist to the Metropolitan Hospital Sunday Fund. There has been no more zealous worker, no more able helper in the direction of accurate figures and precise statements in regard to the London hospitals than Mr. Owthwaite, who has deservedly earned for himself a very high reputation for zeal and earnestness of purpose. No one who really has been in the active life of the hospitals can have failed to recognise his abilities, and all will wish him well for the good services he has rendered. We hope that he will find congenial occupation on his retirement so that his days may be continuously blessed, and bring to Mr. Owthwaite nothing but increasing satisfaction and the best of health.

### A SCOTCH FEVER HOSPITAL VINDICATED.

The Commissioners appointed by the Local Government Board to inquire into the charges of maladministration in the management of the Clackmannan County Combination Fever Hospital have declared that the allegations in the main are unfounded. The medical officer and the committee have been exonerated, and Bailie Strang, the convener of the house committee, has been specially thanked for his services in connection with the inquiry. The hospital, which was established in 1895, contains 52 beds.

### NEW POOR-LAW HOSPITAL CHAIRMAN.

Mr. Fred. W. Blacow has been appointed chairman of the Hope Hospital Committee of the Salford Board of Guardians.

### THE SERIOUS RESULTS OF OVERCROWDING.

Dr. C. H. Phillips, Medical Officer to the Stoke Joint Hospital Board, has revealed in his latest report a large amount of cross infection due to overcrowding in the hospital. He states that nine scarlatina patients contracted diphtheria, while another caught chicken-pox. Owing to wrong notification Dr. Phillips states that twenty-one patients, who were admitted as suffering from diphtheria, contracted scarlatina from patients who had been passed into the diphtheria ward. Further, fifteen scarlatina cases were found on admission to be suffering from chicken-pox, while four patients who had been discharged from the diphtheria ward are stated to have been re-admitted a week later certified as scarlatina. The staff, too, has been affected, four of its members having contracted scarlatina, four enteric, and eight diphtheria. We suppose that his remark that the work has doubled in the last three years while the accommodation remains the same is intended not so much as an excuse as a criticism. At any rate, the moral is the well-known one that in provincial fever hospitals the amount of isolation accommodation, in the form of separate wards, is very notably less than it ought to be.

### FOOTBALL AS A SOURCE OF INCOME

It is doubtful if many institutions have been more fortunate in the help they have received from sport than the Leicester Infirmary. This institution has just received from the Leicester Rugby Football Club a cheque for £111, being the entire proceeds without deduction of the gate and ticket money at the club's final match against Blackheath, which was played at Leicester on April 20. For several years it has been the custom of the club to play a benefit match for the infirmary, and so successful have these proved that the cheque now sent raises the amount given during the past eight years to £1,050. By way of recognition the governors of the infirmary have suggested that another of the prominent members of the committee should be elected a life governor of the institution, thus making the fifth committee-man so honoured.

### THE SCARCITY OF "NORMAL" SCHOOL CHILDREN.

Some astounding statistics concerning the temperature of children inspected in elementary schools are published in the *Lancet* by Dr. Mary Williams. The examination of 1,000 children, not specially selected, showed that only 13.5 per cent. had temperatures below 99° F.; whereas 29.7 per cent. stood at 100° or upwards. These, it must be remembered, were not children supposed to be ill, but attending school and supposed to be well. The author states that she has come to the conclusion that there are few normal children in the elementary schools; she prefers to catalogue them as "not suspected of pulmonary tuberculosis, rheumatism, or tonsillitis," and even then the group includes only 25.4 per cent. of the whole number. The enormous proportion of 24.7 per cent. are said to be actually suffering from pulmonary tuberculosis, and another 16.8 per cent. are suspected of the same disease. We cannot credit the notion that more than 41 per cent. of the youthful proletariat are really the subjects of pulmonary tuberculosis; but if Dr. Williams' figures are accurate it is evident that things are radically wrong.

### COUNTY COUNCIL GRANTS TO HOSPITALS.

Some time ago The Hospital reported that the London County Council had decided to expend in the remaining three months of the financial year a sum not exceeding £100 for contributions to hospitals and convalescent homes in which its employees receive treatment. When the provisional allocation was made, £15 was set aside for contributions to hospitals, but the General Purposes Committee has decided to increase this sum to fifty guineas, and has granted five guineas to each of the hospitals in which employees have been received for treatment. It must be remembered that the benevolent associations formed in the various departments contribute to hospitals, and that the Council's grants are supplementary to these. The Committee argues that it is impossible for the Council's grant to bear any relation to the cost of treatment of its employees, as the employees have a claim in common with other citizens to free treatment. It is proposed to continue the grants and a sum of £500 is to be set aside during the coming year.

### THE LONDON AND COUNTIES MEDICAL PROTECTION SOCIETY.

The annual report of this society is of particular interest this year because in 1911 the society for the first time undertook to pay any damages awarded against its members in a law action defended by the society up to £2,000 per member. This addition to the benefits previously offered to the members entailed the raising of the subscription from ten shillings to a pound. Fortunately for the society, the disbursement required during the year for this purpose amounted to but seventy shillings, and consequently almost the whole of the extra receipts obtained went to swell the accumulated reserve. The work done was of the familiar high standard, and though the financial strength of the society is steadily increasing. The report shows that the society's investments have depreciated by 6 per cent. since they were bought, a proportion which indicates a most unfortunate choice of holdings. Apart from this, the management of the society deserves nothing but commendation.

### THIS WEEK'S DRUG MARKET.

The most important change of the week is a further advance in the price of quinine sulphate, a movement which was anticipated. At the sale of cinchona bark held in Amsterdam last week the prices realised were about 30 per cent. higher than those paid at the preceding sale, and the advance in the price of quinine was announced soon after the result of the bark sale became known. A further rise in the price of quinine is not at all improbable. Citrate of iron and quinine has also advanced. Opium is still inclined towards lower prices, but very little business has been done; in consequence of the weakness of opium, makers of morphine have reduced their prices by sixpence per ounce. Balsam copaiba, balsam tolu, and balsam Peru are dearer. Cocaine is a very dull market, and a fall in price is not unexpected. Oil of cloves is again somewhat dearer in consequence of the further advance in the value of cloves. Cream of tartar is slightly dearer. Ergot is somewhat lower in price. Manna is rather dearer. Gentian root is dearer. Quicksilver is lower. Thymol is dearer.

